# Pediatric critical care capacity in Canada

**DOI:** 10.1093/pch/pxae024

**Published:** 2024-06-07

**Authors:** Saptharishi Lalgudi Ganesan, Daniel Garros, Jennifer Foster, Tanya Di Genova, Patricia S Fontela, Srinivas Murthy

**Affiliations:** Department of Paediatrics & Clinical Neurological Sciences, Schulich School of Medicine & Dentistry, Western University, London; Paediatric Critical Care Unit, Children’s Hospital – London Health Sciences Center, London; Division of Child Health & Therapeutics, Children’s Health Research Institute - Lawson Health Research Institute, London; Division of Pediatric Critical Care, Department of Pediatrics, Faculty of Medicine and Dentistry, University of Alberta, Edmonton; Division of Critical Care, Department of Pediatrics, Pediatric Intensive Care Unit, Stollery Children’s Hospital, Edmonton; Departments of Critical Care and Pediatrics, Faculty of Medicine, Dalhousie University, Halifax; Department of Paediatric Critical Care, IWK Health Centre, Halifax; Department of Pediatrics, Faculty of Medicine & Health Sciences, McGill University, Montreal; Department of Pediatrics, Division of Pediatric Critical Care, Montreal Children’s Hospital, Montreal; Department of Pediatrics, Faculty of Medicine & Health Sciences, McGill University, Montreal; Department of Pediatrics, Division of Pediatric Critical Care, Montreal Children’s Hospital, Montreal; Department of Pediatrics, Faculty of Medicine, University of British Columbia, Vancouver; Pediatric Critical Care, BC Children’s Hospital, Vancouver

**Keywords:** Critical care capacity, Healthcare resources, ICU Resources, Intensive care beds, Pandemic preparedness, Surge capacity

## Abstract

**Objectives:**

Pediatric intensive care unit (PICU) capacity is a current and future health system challenge. Despite experiencing two pandemics in as many decades and surges every winter, we have little to no information on PICU capacity in Canada. Our objective was to characterize the bed capacity of Canadian PICUs and their ability to accommodate surges in demand.

**Methods:**

We conducted a cross-sectional survey to gather information from Canadian PICUs regarding funded/physical beds, unit characteristics, medical staffing, therapies provided, and challenges related to surge management. The survey was completed by a representative from each PICU and validated by PICU Directors. Quantitative survey results were summarized as counts and proportions, while the free-text response was summarized using inductive content analysis.

**Results:**

Representatives from all 19 Canadian PICUs located in 17 hospitals completed the survey and reported having 275 (217 level 3 and 58 level 2) funded beds and 298 physical bed spaces. Of these, 47 beds (35 level 3 and 12 level 2) are in two specialized cardiac PICUs. Roughly 13,385, 13,419, 11,430, and 12,315 children were admitted in the years 2018, 2019, 2020, and 2021, respectively. During a surge, PICUs reported being able to add 5.9 ± 3.4 beds per unit totaling up to 108 temporary surge beds. Several barriers for the successful implementation of surge plans were identified.

**Conclusions:**

Canadian pediatric critical care capacity is comparable to that in many other high-income countries, though our ability to respond to a pandemic/epidemic with significant pediatric critical illness may be limited.

Appropriate assessment and management of critically ill children requires a specialized multidisciplinary healthcare team and dedicated facilities (1,2). Pediatric intensive care units (PICU) have an integral role in managing acute illness, post-operative care, trauma, and complications of chronic diseases in children. However, PICU resources are limited and costly (3).

While a PICU bed includes the bed space, with access to equipment (both monitoring and therapeutic), in healthcare system administration, a “funded PICU bed” includes the physical bed within a unique ecosystem of trained pediatric intensivists, critical care nurses, respiratory therapists, specialized pediatric consulting services, and other members of the interprofessional team (4,5) (Figure 1). Planning for growing and shifting populations, and resource allocation to improve outcomes for Canadian children require an understanding of current PICU capacity in the context of funded PICU beds.

**Figure 1.**
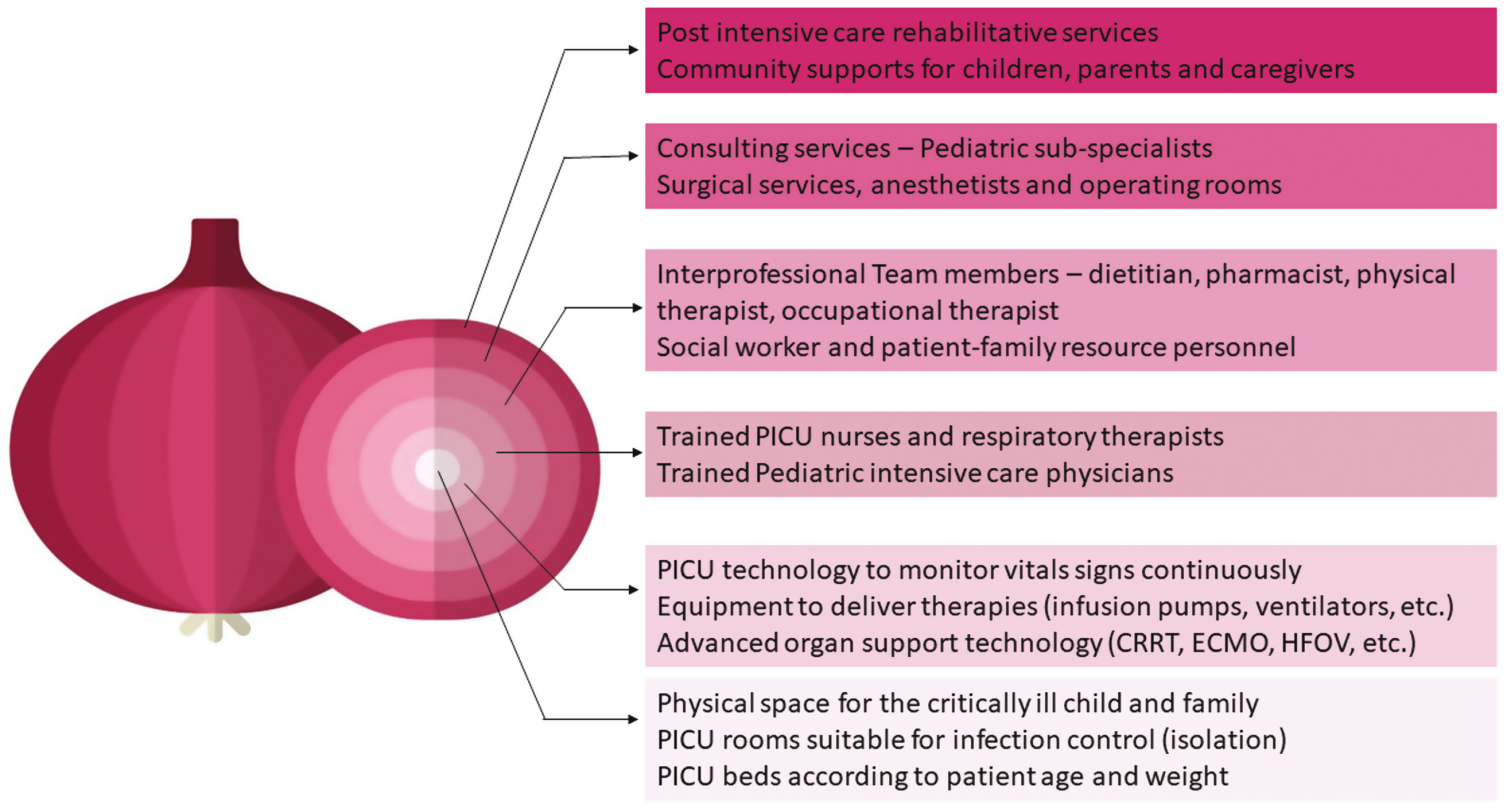
Onion peel model of a “funded” pediatric intensive care unit bed. CRRT Continuous renal replacement therapy; ECMO Extra-corporeal membrane oxygenation; HFOV High frequency oscillatory ventilation

Globally, the pandemic forced healthcare systems to evaluate their critical care capacity and ability to cope with capacity challenges (6–9). While adult ICUs bore the brunt of pandemic-related admissions, the comparatively smaller capacity of pediatric critical care in Canada was also strained (10). Despite few Canadian children with COVID-19 requiring PICU admission, pre-existing capacity limitations were accentuated, leading to strains in service delivery that continue post-pandemic (11). Every year, the seasonal surge of viral infections (12) causes a dramatic strain on capacity across Canada and exposes gaps in our ability to meet demands during surges (13,14). Despite these recent and ongoing challenges, the ability of Canadian PICUs to face surges in demand is largely unknown. Therefore, the objectives of our cross-sectional study were to define the capacity of Canadian PICUs and understand their ability to accommodate surges in demand.

## METHODS

### Study design

We designed an internet-based cross-sectional survey to address the study objectives. The survey design, dissemination, and reporting were carried out in line with the CROSS-reporting guidelines (15).

### Data collection instruments

Physician members from five Canadian PICUs developed a questionnaire to address the following domains: hospital and ICU characteristics, medical staffing, therapies provided, and surge management in the context of a pandemic. The study team participated in an item generation and reduction process to ensure the assessment of each objective. The draft version of the questionnaire was uploaded into the *SurveyMonkey*® platform and was piloted by four PICU physicians from different provinces. The testers completed a sensibility questionnaire to provide feedback regarding the survey questionnaire’s clarity, relevance, face validity, content validity, redundancy, and time for completion. We refined the questionnaire based on the feedback received. The final questionnaire included 18 questions with one question displayed per page.

### Definitions

We defined “PICU” as a unit where fellowship-trained pediatric critical care specialists, and an interprofessional team with PICU-specific skill sets care for critically ill children. We excluded neonatal intensive care units that exclusively treat premature babies and sick term neonates, and adult intensive care units that would occasionally admit children. We adapted the levels of care for PICUs defined by the American Academy of Pediatrics and the Society of Critical Care Medicine to the Canadian context (16–18) to classify PICU beds as “Level 3” if they were designated for children undergoing invasive mechanical ventilation outside of the operating room, emergency room or post-anesthetic care unit. PICU beds were classified as “Level 2” if they were part of a designated step-down or high-dependency unit outside of the general pediatrics floor. “Level 2” PICU beds could provide non-invasive ventilatory support through modalities such as bilevel positive airway pressure.

### Participants

The list of all 19 Canadian PICUs along with email addresses of representatives from each PICU was obtained through the Pediatric subgroup of the Canadian Critical Care Trials Group (CCCTG). An internet-based closed survey was distributed through email to these representatives using a *SurveyMonkey®* link in September 2021 (19). The email inviting them to participate provided information regarding estimated length of time for survey completion as well as the purpose of the survey. The survey was completed by the PICU representatives by December 2021 and the collected data were updated and validated by PICU Directors in September to October 2022. The PICU Directors were sent a private link to a Google sheet containing their unit’s data and asked to review, update, comment on, and validate the data. Whenever clarifications about the responses were needed, the authors contacted the PICU directors and/or representatives. If the survey was not completed, the authors sent reminder emails and contacted the PICU representative via telephone. No incentives were offered for the completion of this survey.

### Analysis

Quantitative survey results were presented as counts, proportions, and standard summary measures. We used the pediatric population (14 years or less) from the 2016 census data (20) to calculate the PICU bed density (beds per 100,000 children) as this is the most relevant category available in the Canadian census. For the calculation of PICU bed density, we used the combined population of provinces/territories that the PICUs serve. We also calculated bed density for the entire population (beds per 100,000) using the Canadian census data (20). To calculate the physician–bed ratio, we divided the number of FTE positions per unit with the number of level 3 beds in the unit. We divided the annual number of admissions in individual units by the number of funded beds (level 3 + 2) to calculate the admission rate per funded bed. Maps showing PICU sites and PICU bed density were generated using the BatchGeo™ (https://batchgeo.com/) and Microsoft Excel™, respectively. For the free text portion of the survey, we used a general inductive content analysis approach (21). We read the answers to a given question, keeping the objective in mind, generated initial categories inductively, then coded each response according to the developing framework, with categories added and adjusted as needed.

## RESULTS

### Canadian PICU capacity

**Figure 2.**
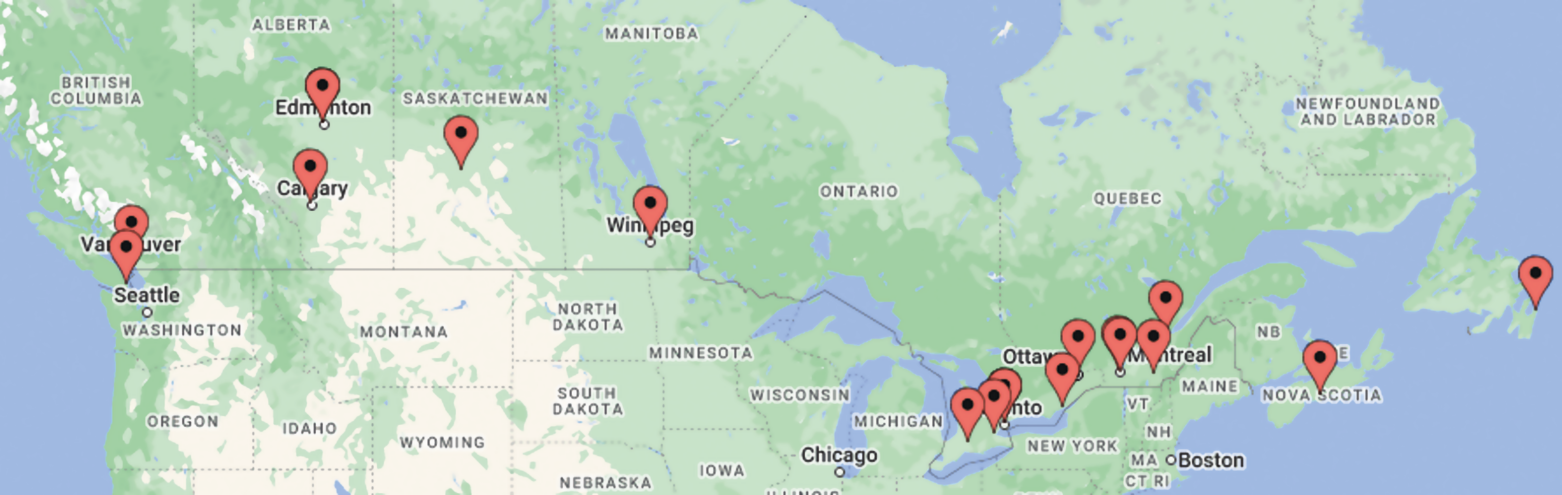
Map location of the 17 Canadian children’s hospitals housing the 19 Pediatric Intensive Care Units (created on *batchgeo.com®*). Montreal children’s hospital and CHU Sainte-Justine children’s hospital are located close to each other and are not distinguishable on this map

**Figure 3.**
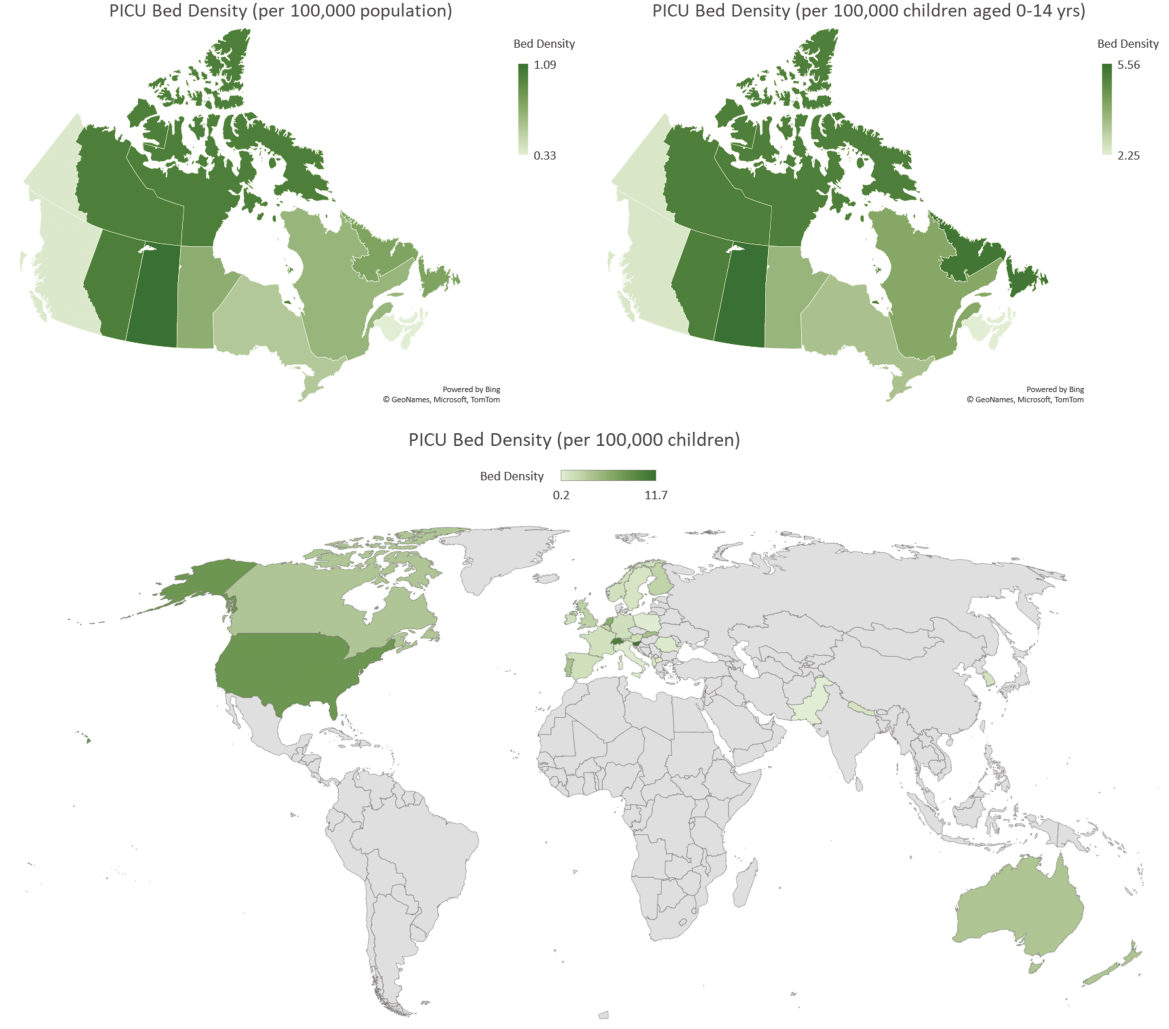
Pediatric intensive care unit bed density. (A) Canadian PICU bed density per 100,000 population; (B) Canadian PICU bed density per 100,000 children aged 0–14 years; (C) global PICU bed density per 100,000 children. European data (survey yr. 2000) derived from Nipshagen et al. (28); Nepal data (yr. 2016) based on Khanal et al. (32); Pakistan data (survey yr. 2009) from Haque et al. (33); South Korea data (survey yr. 2015) based on Yoon JS et al. (23); United States of America data based on Horak RV et al (22). United Kingdom data from the PICANet report (34) (https://www.picanet.org.uk/wp-content/uploads/sites/25/2022/01/PICANet-2021-Annual-Report_v1.0-13Jan2022-2.pdf). Canadian PICU bed density has been calculated based on population of 0–14-year-old children (not 0–18). ANZ data based on that reported in the 2019-20-CCR-Activity-Report (30) (Source: https://www.anzics.com.au/wp-content/uploads/2021/06/2019_20-CCR-Activity-Report.pdf). Nepal PICU bed density based on population of 27.26 million (2016) and children aged 0–14 years old contributing to 32% of Nepalese population in 2016 (Source: https://www.statista.com/statistics/678090/nepal-children-as-a-percentage-of-the-population/)

We invited representatives from all 19 stand-alone PICUs in 17 hospitals (Figure 2) across Canada and received a response from all units (100% completion rate). We received information regarding 217 funded level 3 beds and 58 funded level 2 beds (Table 1). Together, these units reported having 298 physical bed spaces. Two PICUs representing 47 beds (35 Level 3 and 12 Level 2) were specialized cardiac ICUs that cared exclusively for children with medical or surgical cardiac problems. Of the remaining units, 6 were mixed units, caring for children with cardiac and non-cardiac problems while 11 provided care for children with predominantly medical-surgical problems excluding cardiac-surgical conditions. There were no PICUs in Yukon, Nunavut, Northwest Territories (NWT), Prince Edward Island (PEI), and New Brunswick (NB); these provinces were served by PICUs in BC Children’s Hospital (Yukon), CHEO (Baffin Island, Nunavut), Stollery Children’s and Alberta Children’s (NWT), and IWK Health (PEI and NB). The province with the highest PICU bed (medical-surgical and cardiac) density was Saskatchewan (5.56 beds/100,000 children), while the provinces with the lowest PICU bed density were Nova Scotia, Prince Edward Island & New Brunswick (2.24 beds/100,000 children) (Figure 3). A total of 133.3 funded full-time-equivalent (FTE) positions were filled by fully trained pediatric critical care physicians. The ratio of FTE physician-per-funded level three beds across the country was 0.63 ± 0.14.

**Table 1.** Canadian PICU characteristics—beds, physician staffing, services, isolation, and surge capacity

PICU location	Type	Funded Level 3 beds	Funded Level 2 beds	Funded physician FTEs	ECMO availability	Surge beds	Isolation beds (%)	Retrieval team
British Columbia								
Vancouver	Combined	12	0	10	Yes	12	76–100	Yes
Victoria	Medical-Surgical	5	0	2.75	No	3	26–50	No
Alberta								
Calgary	Medical-Surgical	11	4	10	Yes	3	76–100	Yes
Edmonton	Cardiac-Surgical	15	8	8	Yes	16	76–100	Yes
Edmonton	Medical-Surgical	15	12	8	Yes	10	76–100	Yes
Saskatchewan								
Saskatoon	Medical-Surgical	12	0	5.5	No	6	26–50	Yes
Manitoba								
Winnipeg	Medical-Surgical	9	0	5	No	6	76–100	Yes
Ontario								
London	Medical-Surgical	12	2	6	No	4	51–75	Yes
Hamilton	Medical-Surgical	12	4	6	No	4	76–100	Yes
Toronto	Medical-Surgical	20	12	12	Yes	5	26–50	Yes
Toronto	Cardiac-Surgical	20	0	8	Yes	0	26–50	Yes
Kingston	Medical-Surgical	0	4	1	No	6	76–100	No
Ottawa	Combined	10	6	7	Yes	5	76–100	Yes (up to 3 y)
Quebec								
Montreal Children’s	Combined	12	6	9	Yes	0	51–75	No
CHU St. Justine	Combined	24	0	14	Yes	14	76–100	No
Quebec City	Combined	12	0	9	Yes	4	0–25	No
Sherbrooke	Medical-Surgical	6	0	5	No	4	51–75	Yes
Atlantic region								
St. John’s	Medical-Surgical	4	0	3	No	4	26–50	No
Halifax	Combined	6	0	4	Yes	9	0–25	Yes
Total	–	217	58	133.25	–	108	–	–

*PICU Pediatric intensive care unit; ECMO Extra-corporeal membrane oxygenation; FTE Full-time equivalent*

### Population served

**Figure 4.**
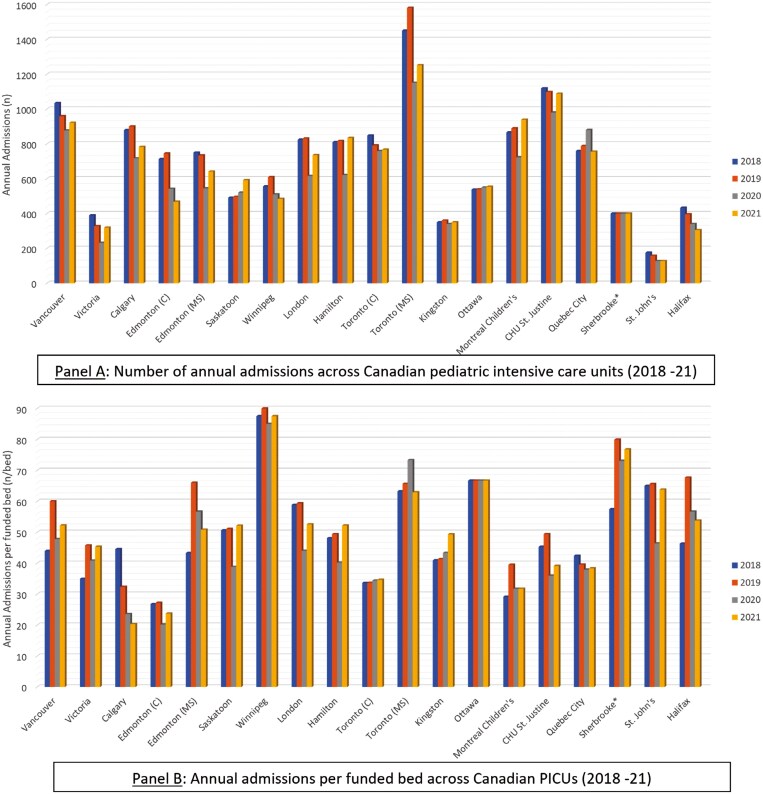
Number of annual admissions and annual admissions per funded bed across Canadian PICUs (2018–2021)

In the years 2018 to 2021, the 19 PICUs admitted roughly 13,385, 13,419, 11,430, and 12,315 Canadian children, respectively. One PICU provided rough estimates (~400 per year) regarding their annual admissions. Annual admissions and annual admissions per funded bed over these 4 years have been illustrated in Figure 4A and 4B, respectively. All 19 PICUs cared for children aged 1 month to 16 years of age; 16 PICUs cared for children up to 18 years old and 4 for young adults aged 19 to 20 years on a case-to-case basis. Neonates (0–28 days of life) were admitted in 18 out of 19 PICUs under the following circumstances: Neonate discharged home after birth and returns with critical illness (n = 10), neonates with congenital cardiac lesions (n = 8), neonates requiring CRRT or ECMO (n = 6), and neonates with surgical problems (n = 3). One of the 19 units cared for critically ill peri-partum women. Seventeen PICUs provided care for children with polytrauma and/or neurosurgical problems, 16 for children with severe burns, and 8 offered peri-operative care for solid organ transplant recipients.

### Services offered

Seventeen PICUs offered continuous electroencephalography (29). All 19 units reported access to inhaled nitric oxide therapy while 18 offered high-frequency oscillator ventilation (HFOV). Nine PICUs offered extra-corporeal membrane oxygenation (ECMO) and 18 offered continuous renal replacement therapy (CRRT). Of the 19 PICUs, 11 accepted children transferred from other PICUs for advanced therapies. Both cardiac PICUs offered ventricular assist device (VAD) support.

Children with infectious diseases that are airborne or spread through droplets require admission to a room with isolation capabilities. Two PICUs reported that 0% to 25% of beds were isolation beds; four PICUs had 26% to 50% beds that can hold patients who require isolation; three PICUs stated that 51% to 75% beds had isolation capabilities while the remaining 10 units were able to hold patients requiring isolation in 76% to 100% of their beds (Table 1).

### Surge management

All 19 PICUs reported having a surge management plan. PICUs reported being able to add 5.9 ± 3.4 (range: 0–14) temporary surge beds, together creating 108 additional temporary surge beds (Level 3 + Level 2). In a surge situation, respondents indicated that non-urgent cardiac and other surgical procedures could be completely halted to divert existing beds to care for critically ill children; this could potentially free up about 70% beds in cardiac-surgical PICUs (32 beds) and 20% beds in medical-surgical PICUs (44 beds), considering that emergency life-saving cardiac and non-cardiac surgeries would need to continue. Barriers for the successful implementation of surge plans have been summarized with illustrative quotes, themes derived from the responses and emerging narratives in Table 2.

**Table 2.** General inductive content analysis of challenges to mounting an effective surge response as identified by Canadian PICUs

Category (n)	Representative quotes	Narrative description
Shortage of human resources(n = 17)	“We have 6 physical beds, and, on most days, we only have 3–4 nurses on the schedule. Problems will arise if the skeleton nursing staff become ill necessitating staying home. We will be relying on overtime and potentially nurses who no longer work in the PICU being re-deployed back.” “We have space and equipment to operate at 120–140% capacity but human resources will be the major issue.”	Lack of trained personnel, including critical care nurses, respiratory therapists, and physicians to staff these additional or temporary surge beds could be the most important “bottleneck” in mounting a surge response
Limited number of beds (n = 6)	“We are often struggling to find a PICU bed when all our physical beds are occupied. We spend many hours on the phone trying to connect with other PICUs in the province, in the neighboring provinces and sometimes, even outside Canada to find a PICU bed for the critically ill child.” “Ward capacity is limited to accommodate PICU discharges and ED admissions.”	Shortage of beds in the PICU and the Pediatric wards keep some children waiting for a PICU bed and others in the PICU longer than necessary
Supporting adult patients (n = 2)	“There is also ongoing collaboration with the adult critical care such that there are adults being cared for in the pediatric ICU which is not yet set for an end date.”	A surge situation increasing critical care requirements for adults and children will redirect PICU resources to support adult patients
Staffing attrition (n = 2)	“Our RN staffing is much more challenged than previous due to burn-out, mat leaves, retirements, vaccine refusals and people deciding to leave the organization for various other reasons.”	PICU providers are experiencing an unprecedented attrition in numbers
Ramping down surgical procedures (n = 1)	“Our surge plan involved our PACU, but this may not be feasible if we don’t ramp down ORs like happened previously.”	Surgeries would have to be cancelled to allow PICUs to handle surge and this may not always be possible
Changing demographic of PICU admissions (n = 1)	“Children with complex medical comorbidities requiring frequent PICU admissions seem to be growing in numbers.”	A higher proportion of critically ill children have medical comorbidities and have complex needs, and are staying in the PICU longer
Staffing models (n = 1)	“Lack of governmental understanding that PICUs can’t just ramp up their occupancy or the amount of work they do and looking at the average census doesn’t allow us to staff for a surge.”	PICUs needs to reimagine their staffing models to be able to tackle surge situations

“n” refers to the number of Canadian PICUs reporting challenges in the specified category

## DISCUSSION

This study characterizes the national capacity and surge management capabilities of Canadian PICUs. We found substantial variation in existing PICU capacity as well as varied ability to accommodate surges in pediatric critical care admissions across Canada. Such variation in current and surge capacity could compromise equity and result in differential decision-making about access to PICU care, and the services delivered during times of increased demand (5).

Like other countries, Canada’s PICUs are regionalized within larger urban areas (Table 3) (22–24,26–28,30–33). Our PICU bed-population ratio is comparable to that in Europe (historical data; yr. 2000) (28), UK (25,34), and Korea (23). The USA is an outlier with its 2016 PICU bed density comparable to the combined pediatric and adult ICU bed density in Canada of 9.5 beds per 100,000 (5,27). In comparison to the USA (22), Canada does not have specialized PICUs solely focused on care of children with polytrauma or neurological problems. Our work collected data on “funded beds” that are fully operationalized as of September to October 2022, and “physical beds” that have the necessary equipment to support a mechanically ventilated critically ill child but have not been staffed yet (35). In addition, the use of pediatric population (14 years or less) from the 2016 census data as the denominator is likely to have overestimated the bed density.

**Table 3.** Pediatric critical care capacity estimates for Canada in comparison to other regions/countries

	Canada (2022)	ANZ (30) (2020)	Europe (28) (2000)	USA (22) (2016)	United Kingdom* (34) (2020)	Spain (24) (1996)	Pakistan (33) (2009)	Nepal (32)(2016)	South Korea (23) (2015)
Number of PICUs	19	13	144	344	32	34	16	18	13
Type	(n = 19)		(n = 104)			(n = 31)		(n = 16)	
Pediatric	19	9	92	344	--	18	--	--	12
Neonatal + Pediatric	0	1	8	0	--	12	--	--	0
Adult + Pediatric	0	3	4	0	--	1	--	--	1
PICU subtype									
Medical-surgical	11	--	--	285	--	--	12	--	9
Cardiac	2	--	--	49	--	--	3	--	3
Mixed	6	--	--	--	--	--	1	--	1
Trauma	0	--	--	2	--	--	--	--	--
Neuro	0	--	--	8	--	--	--	--	--
PICU beds, n	217	213^§^	1198	5908	369	271	155	93	113
PICUs with									
6 or fewer beds	5	--	21	--	--	15	--	--	--
7–12 beds	7	--	36	--	--	8	--	--	--
13 or more beds	7	--	35	--	--	8	--	--	--
Funded/staffed beds per PICU	11.4 ± 5.9	11.5^║^	9 (2–46)	12 [8,20]	--	--	9.7 (4–28)	5 [5–6]	9.4 (2–30)
PICU beds per 100,000 children	3.72^‡^	3.8	2.7 (0.5–11.7)	8	2.7 (33)–2.9^†^	--	0.2	1.1^#^	1.3
Admissions per year	12,296^**^	8972	--	--	16,429	9585	7376	--	--

^*^PICANet report includes data from UK and Ireland (https://www.picanet.org.uk/wp-content/uploads/sites/25/2022/01/PICANet-2021-Annual-Report_v1.0-13Jan2022-2.pdf);

^†^Based on national level population estimates by year, age and UK country - https://statswales.gov.wales/catalogue/population-and-migration/population/estimates/nationallevelpopulationestimates-by-year-age-ukcountry;

^‡^Calculated based on population of 0–14-year-old children;

^§^Physical beds as reported in the 2019-20-CCR-Activity-Report;

^║^Based on available beds (Source: https://www.anzics.com.au/wp-content/uploads/2021/06/2019_20-CCR-Activity-Report.pdf);

^#^Calculated based on Nepal population of 27.26 million (2016) and children aged 0–14 years old contributing to 32% of Nepalese population in 2016 (Source: https://www.statista.com/statistics/678090/nepal-children-as-a-percentage-of-the-population/);

^**^Sherbrooke provided approximate census data. Kingston does not have level 3 PICU beds (ventilated) and therefore their census was also not included

Although all Canadian PICUs reported having a crisis/surge response plan, they may require further investment for dealing with such situations (Table 2). Four barriers stood out—Firstly, all PICUs reported a shortage of trained personnel such as critical care nurses and respiratory therapists to staff surge beds. In addition, PICUs are facing challenges staffing existing funded beds due to significant attrition of existing frontline staff, which could be a result of burnout, retirement, relocation to non-ICU areas, etc. (36). Secondly, at least six centers reported that they needed more physical bed spaces in the PICUs and downstream to accommodate surge. Thirdly, it was reported that a sizable proportion of PICU beds are not set up to isolate patients with transmissible infections and may require redesigning of the physical layout. Inability to implement appropriate infection control practices could lead to outbreaks and contamination of healthcare workers, further straining human resources during a surge and risk worsening clinician stress/ burnout (37,38). Lastly, during the peak of COVID-19 pandemic, the Canadian adult critical care system was able to accommodate 150% to 200% of pre-pandemic averages by deferring elective surgeries/procedures, funding new ICU beds, identifying temporary surge spaces, redeploying staff (some from PICUs), and introducing team-based models of care (39). Current PICU surge management plans may not permit similar capacity expansion.

We also learnt through this study that, in contrast to other countries with nationally coordinated registries of critically ill children (30,34), data on PICU occupancy, diagnosis, or case mix in Canada are only shared within each province. Availability of real-time regarding PICU bed availability, resource utilization, and de-identified data on patient case mix and outcomes would allow effective, real-time decision-making regarding resource allocation and efficient interprovincial collaboration. This would also allow streamlining of interprovincial transport processes and a federally coordinated interprovincial healthcare model during pandemic surges. Without a coordinated central database, PICUs in Canada will continue working in isolation, lacking coordination and situational awareness to respond in times of crisis.

Our study has important limitations. First, reporting of capacity was done by one representative and validated by the clinical directors. Though we carefully requested exact numbers and checks, reported and actual capacity may not be the same (19). Second, we did not explore daily census/occupancy trends, which are valuable but harder to collect. Studies show that ICUs that consistently operate in a high occupancy state (85–100%) have a 19% higher adjusted patient mortality risk (40). Third, PICU bed and staffing capacity are in continuous flux and our cross-sectional snapshot may not capture the day-to-day changes. However, we were careful in requesting such information and we believe that our surge capacity was well-reported by the respondents. Our data presents the best scenario for each unit when all funded beds can be staffed. The actual capacity would be lower if the unit is unable to staff all funded beds. Fourth, the PICU census has a significant seasonal variation with peaks during winter months (41,42), however, our study did not explore seasonal variations in PICU capacity. PICU bed funding as well as staffing must be planned so that patient care does not suffer, and staff are not subjected to unsafe working environments during periods of seasonal surge in admissions. Therefore, future studies should explore day-to-day census and operational aspects of Canadian PICUs, as well as the feasibility of quickly training nurses, respiratory therapists, and other interprofessional team members from non-PICU areas. Lastly, the capacity data was validated and updated in September to October 2022. Therefore, changes to capacity in response to the 2022 winter surge would not be captured in our work. Despite these limitations, our work is essential to raise awareness about the ongoing limitations in pediatric critical care capacity, as well as surge capacity, in Canada.

In conclusion, Canadian pediatric critical care capacity was comparable to that in many high-income countries, though our ability to respond to a pandemic/epidemic surge with significant pediatric critical illness may be limited. Federal and provincial governments should collaboratively and proactively plan sustainable increases in long-term Canadian PICU capacity including provision for centralized data coordination and improved patient isolation.

## Data Availability

The datasets used and/or analyzed during the current study are available from the corresponding author on reasonable request.

## References

[1] Levin DL , Downes JJ, Todres ID. History of pediatric critical care medicine. J Pediatr Intensive Care 2015;2:147–67. 10.3233/PIC-13068 PMC6530732 31214438

[2] Frankel L , Hsu B, Yeh T, et al Criteria for critical care infants and children: PICU admission, discharge, and triage practice statement and levels of care guidance. Pediatr Crit Care Med 2019;20(9):847–87. 31483379 10.1097/PCC.0000000000001963

[3] Opgenorth D , Stelfox H, Gilfoyle E, et al Perspectives on strained intensive care unit capacity: A survey of critical care professionals. PLoS One 2018;13(8):e0201524. 30133479 10.1371/journal.pone.0201524 PMC6104911

[4] Duggal A , Orsini E, Mireles-Cabodevila E, et al Surge capacity and capability of intensive care units across a large healthcare system: An operational blueprint for regional integration. Am J Disaster Med 2021;16(3):179–92. doi: https://doi.org/10.5055/ajdm.2021.0400 34904702

[5] Fowler R , Abdelmalik P, Wood G, et al Critical care capacity in Canada: Results of a national cross-sectional study. Crit Care 2015;19(1):133. 25888116 10.1186/s13054-015-0852-6 PMC4426537

[6] Ma X , Vervoort D. Critical care capacity during the COVID-19 pandemic: Global availability of intensive care beds. J Crit Care 2020;58:96–7. doi: https://doi.org/10.1016/j.jcrc.2020.04.012 32408107 PMC7194590

[7] Carenzo L , Costantini E, Greco M, et al Hospital surge capacity in a tertiary emergency referral centre during the COVID-19 outbreak in Italy. Anaesthesia 2020;75(7):928–34. 32246838 10.1111/anae.15072

[8] Yamamoto T , Ozaki M, Kasugai D, Burnham G. Assessment of critical care surge capacity during the COVID-19 pandemic in Japan. Health Secur 2021;19(5):479–87. doi: https://doi.org/10.1089/hs.2020.0227 34346775 PMC10818035

[9] Montgomery J , Stokes-Lampard H, Griffiths M, Gardiner D, Harvey D, Suntharalingam G. Assessing whether COVID-19 patients will benefit from critical care, and an objective approach to capacity challenges during a pandemic: An Intensive Care Society clinical guideline. J Intensive Care Soc 2021;22(3):204–10. doi: https://doi.org/10.1177/1751143720948537 34422102 PMC8373279

[10] Zee-Cheng J , McCluskey C, Klein M, et al Changes in pediatric ICU utilization and clinical trends during the coronavirus pandemic. Chest 2021;160(2):529–37. 33727033 10.1016/j.chest.2021.03.004 PMC7954775

[11] Drouin O , Hepburn CM, Farrar DS, et al Characteristics of children admitted to hospital with acute SARS-CoV-2 infection in Canada in 2020. CMAJ 2021;193(38):E1483–93. 34580141 10.1503/cmaj.210053 PMC8486480

[12] Health GoC-P. Respiratory Virus Report, Week 45 - ending November 12, 2022 - Canada.ca canada.ca2022 (updated 2022-11-17). https://www.canada.ca/en/public-health/services/surveillance/respiratory-virus-detections-canada/2022-2023/week-45-ending-november-12-2022.html (accessed June 23, 2023).

[13] Pelley L. ANALYSIS | Why are respiratory viruses like RSV hitting Canadian kids so hard this year? CBC News. 2022.

[14] Weeks C. Children’s hospitals are overwhelmed across Canada. Experts weigh in on what’s to blame – and what’s not. The Globe and Mail. 2022.

[15] Sharma A , Minh Duc N, Luu Lam Thang T, et al A consensus-based checklist for reporting of survey studies (CROSS). J Gen Intern Med 2021;36(10):3179–87. 33886027 10.1007/s11606-021-06737-1 PMC8481359

[16] Rosenberg D , Moss M; American Academy of Pediatrics SoCC. Guidelines and levels of care for pediatric intensive care units. Pediatrics 2004;114(4):1114–25. 15466118 10.1542/peds.2004-1599

[17] Stark A ; American Academy of Pediatrics CoFN. Levels of neonatal care. Pediatrics 2004;114(5):1341–7. 15520119 10.1542/peds.2004-1697

[18] Randolph A , Gonzales C, Cortellini L, Yeh T. Growth of pediatric intensive care units in the United States from 1995 to 2001. J Pediatr 2004;144(6):792–8. doi: https://doi.org/10.1016/s0022-3476(04)00218-5 15192628

[19] Foster J , Lee L, Seabrook J, et al Family presence in Canadian PICUs during the COVID-19 pandemic: a mixed-methods environmental scan of policy and practice. CMAJ Open 2022;10(3):E622–32. 10.9778/cmajo.20210202 PMC9262350 35790228

[20] Canada SCS. Census Profile, 2016 Census [Government Website]. Government of Canada; 2017 (updated June 18, 2019). https://www12.statcan.gc.ca/census-recensement/2016/dp-pd/prof/index.cfm?Lang=E(accessed May 12, 2023).

[21] Thomas DR. A General inductive approach for analyzing qualitative evaluation data. Am J Eval 2006;27(2):237–46. doi: https://doi.org/10.1177/1098214005283748

[22] Horak R , Griffin J, Brown A, et al Growth and changing characteristics of pediatric intensive care 2001-2016. Crit Care Med 2019;47(8):1135–42. 31162205 10.1097/CCM.0000000000003863

[23] Yoon J , Jhang W, Choi Y, et al Current status of pediatric critical care in Korea: results of 2015 National Survey. J Korean Med Sci 2018;33(49):e308. 30505252 10.3346/jkms.2018.33.e308 PMC6262189

[24] López-Herce J , Sancho L, Martinón J. Study of paediatric intensive care units in Spain. Spanish Society of Paediatric Intensive Care. Intensive Care Med 2000;26(1):62–8. 10663282 10.1007/s001340050013

[25] Sinha R , Aramburo A, Deep A, et al Caring for critically ill adults in paediatric intensive care units in England during the COVID-19 pandemic: planning, implementation and lessons for the future. Arch Dis Child 2021;106(6):548–57. 33509793 10.1136/archdischild-2020-320962

[26] Muttalib F , González-Dambrauskas S, Lee J, et al Pediatric emergency and critical care resources and infrastructure in resource-limited settings: a multicountry survey. Crit Care Med 2021;49(4):671–81. 33337665 10.1097/CCM.0000000000004769

[27] Odetola F , Clark S, Freed G, Bratton S, Davis M. A national survey of pediatric critical care resources in the United States. Pediatrics 2005;115(4):382–6. 10.1542/peds.2004-1920 15805338

[28] Nipshagen M , Polderman K, DeVictor D, Gemke R. Pediatric intensive care: result of a European survey. Intensive Care Med 2002;28(12):1797–803. doi: https://doi.org/10.1007/s00134-002-1532-y 12447526

[29] LaRovere K , Riggs B, Poussaint T, et al Neurologic involvement in children and adolescents hospitalized in the United States for COVID-19 or multisystem inflammatory syndrome. JAMA Neurol 2021;78(5):536–47. 33666649 10.1001/jamaneurol.2021.0504 PMC7936352

[30] Australia New Zealand Intensive Care Society. Paediatric Activity Report - Paediatric Annual/Activity Reports, ANZICS 2018. 2018. https://www.anzics.com.au/annual-reports/ (accessed November 14, 2023).

[31] Murthy S , Leligdowicz A, Adhikari N. Intensive care unit capacity in low-income countries: a systematic review. PLoS One 2015;10(1):e0116949. 25617837 10.1371/journal.pone.0116949 PMC4305307

[32] Khanal A , Sharma A, Basnet S. Current state of pediatric intensive care and high dependency care in Nepal. Pediatr Crit Care Med 2016;17(11):1032–40. doi: https://doi.org/10.1097/PCC.0000000000000938 27679966

[33] Haque A , Ladak LA, Hamid MH, Mirza S, Siddiqui NR, Bhutta ZA. A national survey of pediatric intensive care units in Pakistan. J Crit Care Med 2014;842050. doi:10.1155/2014/842050.

[34] Leicester UoLa. PICANet - Paediatric Intensive Care Audit Network 2022. https://www.picanet.org.uk/ (accessed January 23, 2023).

[35] Payne E. Provincial investment will nearly double CHEO’s ICU and critical care beds. Ottawa Citizen. 2022. Provincial investment will nearly double CHEO's ICU and critical care beds | Ottawa Citizen (accessed January 14, 2023).

[36] Orrù G , Marzetti F, Conversano C, et al Secondary traumatic stress and burnout in healthcare workers during COVID-19 outbreak. Int J Environ Res Public Health 2021;18(1):337. doi: https://doi.org/10.3390/ijerph18010337 33466346 PMC7794988

[37] Magnavita N , Chirico F, Garbarino S, Bragazzi N, Santacroce E, Zaffina S. SARS/MERS/SARS-CoV-2 outbreaks and burnout syndrome among healthcare workers. an umbrella systematic review. Int J Environ Res Public Health 2021;18(8):4361. doi:10.3390/ijerph18084361 33924026 PMC8072681

[38] Dryden-Palmer K , Moore G, McNeil C, et al Moral distress of clinicians in Canadian Pediatric and Neonatal ICUs. Pediatr Crit Care Med 2020;21(4):314–23. 31725530 10.1097/PCC.0000000000002189

[39] Aziz S , Arabi Y, Alhazzani W, et al Managing ICU surge during the COVID-19 crisis: rapid guidelines. Intensive Care Med 2020;46(7):1303–25. 32514598 10.1007/s00134-020-06092-5 PMC7276667

[40] Wilde H , Mellan T, Hawryluk I, Dennis JM, Denaxas S, Pagel C, et al The association between mechanical ventilator availability and mortality risk in intensive care patients with COVID-19: a national retrospective cohort study. 2021. 10.1186/s12916-021-02096-0 PMC8404408 34461893

[41] Andrews C , Maxwell S L, Kerns E, McCulloh R, Alverson B. The association of seasonality with resource use in a large national cohort of infants with bronchiolitis. Hosp Pediatr 2021;11(2):126–34. 33436417 10.1542/hpeds.2020-0120 PMC7831374

[42] O’Donnell D , Parslow R, Draper E. Deprivation, ethnicity and prematurity in infant respiratory failure in PICU in the UK. Acta Paediatr 2010;99(8):1186–91. doi: https://doi.org/10.1111/j.1651-2227.2010.01803.x 20236254

